# How human–machine collaboration fuels employees' digital creativity: the serial mediating roles of technology acceptance and positive emotion

**DOI:** 10.3389/fpsyg.2026.1861091

**Published:** 2026-06-23

**Authors:** Lijun Xia, Zhouyan Zheng, Binlin Si, Huabin Wu, Jiali Zhu

**Affiliations:** 1 Zhejiang University of Finance and Economics Dongfang College, Haining, China; 2 School of Business, Nanjing University, Nanjing, China

**Keywords:** employee digital creativity, human–machine collaboration, job demands-resources (JD-R) theory, positive emotion, technology acceptance

## Abstract

Against the backdrop of AI-driven digital transformation, digital creativity has emerged as a critical driver of corporate innovation. Consequently, investigating the mechanisms through which human–machine collaboration influences employees‘ digital creativity holds profound theoretical and practical significance. Grounded in the Job Demands-Resources (JD-R) theory, this study constructs a structural equation model to explore these underlying pathways, specifically examining the chain-mediating roles of technology acceptance and positive emotion. Using cross-sectional multi-source survey data from 401 employees, empirical findings reveal that human–machine collaborative behavior shows a significant positive association with employee digital creativity. Furthermore, technology acceptance serves as a primary cognitive channel, playing a crucial mediating role in this relationship. Collaboration with AI also indirectly fosters digital creativity by cultivating positive emotions, which supply the psychological momentum necessary for creative endeavors. Notably, the results confirm a serial mediation pathway, establishing the sequential pattern: “human–machine collaboration → technology acceptance → positive emotions → employees' digital creativity.” Ultimately, this study expands the scope of the JD-R theory by enriching its conceptualization of job resources in the digital era, while simultaneously offering multidimensional practical insights for leveraging human–machine collaboration to empower workforce creativity during enterprise intelligent transformation.

## 1 Introduction

Society is currently experiencing a profound digital transformation propelled by artificial intelligence (AI). AI technology has transitioned from theoretical frameworks to practical applications, deeply integrating into the workflows of various organizations and significantly impacting how workers complete tasks, solve problems, and engage in innovative thinking. As digital innovation permeates our daily lives, creativity takes on a new form: digital creativity (Lee, 2015). In this context, whether the substantial

investments companies make in AI technology can truly translate into innovative outcomes depends heavily on whether employees, through their interactions with AI, can enhance work engagement to improve efficiency and deliver creative digital results (Bagea et al., 2024). This is particularly relevant as digital transformation fundamentally alters business model innovation, necessitating a reevaluation of how value is created and captured across industries (Vaska et al., 2021).

Human–machine collaboration, acting as a strategic model for enterprise design and innovation (Song et al., 2024), encompasses human intelligence, artificial intelligence, and their synergistic interactions. This holds significant theoretical and practical importance for employees seeking to realize self-worth and unleash digital creativity. This significance stems not only from enhanced operational efficiency but also from the potential to transform the innovation paradigm by merging AI's computational and data-processing powers with human intuition, critical thinking, and contextual comprehension.

From a strategic business perspective, continuous innovation is the primary source of core competitiveness in rapidly evolving markets (Nica et al., 2017). The human–machine collaboration model transforms AI technology from a labor-substituting automation tool into an empowering partner that augments human capabilities and opens new paths for corporate employees to create value. Consequently, this “empowerment” of employees' digital creativity directly impacts whether enterprises can effectively convert technological investments into sustainable innovation advantages. Furthermore, modern enterprise strategies surrounding technology implementation not only dictate performance but also have a profound impact on employee psychological satisfaction, making it vital to manage these transitions carefully (Ren and Zhong, 2022).

At the individual employee level, the advent of the digital age presents new challenges to workers' skills and values (Rion and Any, 2025). However, human–machine collaboration charts a developmental path of “symbiotic evolution” for employees, transitioning them from passive operators of technology to proactive decision-makers who harness its potential (Rahayu et al., 2024). When employees effectively leverage AI tools to expand their cognitive boundaries, delegate repetitive tasks to machines, and focus more on high-value creative ideation, their work autonomy and sense of achievement are significantly enhanced (Parent-Rocheleau and Parker, 2022). Similar to how the strategic use of digital information search processes has been shown to foster creative question generation (Mao et al., 2022; Oldham and Da Silva, 2015), employees in the workplace can leverage AI to navigate vast amounts of data and elevate their creative outputs. In addition to fostering intrinsic motivation and digital innovation, this approach plays a crucial role in helping workers reevaluate their own value and attain professional sustainability in the digital age.

Therefore, investigating the effect of human–machine collaboration on employees‘ digital creativity has emerged as a research topic of immense theoretical depth and practical significance. A review of the literature reveals that many studies have concentrated on how external factors affect employee creativity. Examples include the positive effects of social media (Korzynski et al., 2020), digital leadership (Zhu et al., 2022), and workplace environments (Amabile et al., 1996). While the literature frequently explores mediating mechanisms—such as how team resilience and efficacy predict creativity (Fan et al., 2021)—the specific cognitive and emotional pathways bridging human-machine collaboration and creativity remain underexplored. Existing studies have predominantly examined the influence of individual employee characteristics (Shalley et al., 2004), leaving a noticeable gap regarding employees' cognitive and affective processes.

According to Venkatesh et al. (2003), studies based on the Technology Acceptance Model (TAM) highlight the significance of cognitive assessment, suggesting that employees' acceptance of AI technology is the foundational basis for their usage behavior. Conversely, positive psychology emphasizes how positive emotions promote exploratory behavior and broaden cognitive limits (Fredrickson, 2001). The positive influence of human–machine collaboration on digital creativity cannot be achieved through mere technical deployment; its essence lies in understanding and managing the micro-level psychological mechanisms in human–AI interactions (Raisch and Krakowski, 2021). Indeed, recent evidence highlights that users' acceptance of AI systems is a highly complex process driven by a combination of functionality and social emotions (Zhang et al., 2021). If AI technology systems fail to gain employees' cognitive recognition and emotional acceptance, they may become costly and ineffective.

In summary, the current research gap lies in the absence of an integrated, dual-path chain mediation model that combines cognitive and affective pathways to reveal the intrinsic psychological mechanisms through which human–machine collaboration influences employees‘ digital creativity. To close this gap, this work builds a chain mediation model based on the Job Demands-Resources (JD-R) theory (Bakker and Demerouti, 2014). By treating human-machine collaboration as a critical job resource, this study aims to delve into the internal mediating roles of technology acceptance and positive emotions in the relationship between human–machine collaboration and employees' digital creativity.

## 2 Research hypotheses

### 2.1 Human–machine collaboration and employees' digital creativity

This study anchors its theoretical framework in the job demands-resources (JD-R) theory (Bakker and Demerouti, 2014). Since its inception, this framework has evolved into a prominent lens for elucidating employee psychological and behavioral dynamics in contemporary work settings. The JD-R model posits that job characteristics bifurcate into two overarching categories: job demands—physical, psychological, social, or organizational aspects of the job that require sustained effort and incur physiological or psychological costs—and job resources—the functional means that facilitate work goal attainment, reduce job demands, or stimulate personal growth and development (Schaufeli, 2015). Within this framework, human–machine collaboration (HMC) is conceptualized as an emerging socio-technical job resource, distinct from mere technology exposure or instrumental AI usage, that encompasses the complementary interdependence and coordinative integration between human cognitive capabilities (creativity, contextual reasoning, ethical judgment) and machine computational strengths (data processing, pattern detection, automation) (Seeber et al., 2020; Dellermann et al., 2021). HMC transcends the frequency-based conceptualization of AI usage, which merely captures the intensity of technology interaction (Baird and Maruping, 2021) and the implementation-focused notion of digital tool adoption, which emphasizes assimilation into work routines (Venkatesh et al., 2003). Instead, HMC captures the relational dynamics of task complementarity and mutual adjustment wherein humans and AI agents engage in joint problem-solving through differentiated yet coordinated contributions (Wilson and Daugherty, 2018; Raisch and Krakowski, 2021). As a job resource, HMC is theorized to activate digital creativity through dual pathways. First, HMC provides cognitive-augmentation resources: intelligent systems process voluminous data, identify non-obvious patterns, and generate analytical outputs, thereby extending employees' cognitive boundaries and enabling them to transcend traditional information-processing limitations (Hemmer et al., 2025). This augmentation expands the scope of creative ideation by furnishing novel inputs and computational capabilities that complement human imaginative capacities. Second, operating through the resource-gain principle (Bakker and Demerouti, 2017), HMC automates routine, low-complexity tasks, thereby liberating employees' psychological and temporal resources for higher-order cognitive activities—namely, creative exploration and experimentation. This resource-depletion prevention mechanism preserves the mental energy requisite for creative engagement. Direct activation of goal-directed behavior. In accordance with the JD-R proposition that job resources can directly stimulate motivational processes without necessitating mediators (Schaufeli, 2015), HMC by enhancing employees' sense of competence and autonomy in decision-making (Gagné and Deci, 2005), directly galvanizes intrinsic motivation and, consequently, digital creativity. Employees who perceive themselves as capable of effectively leveraging AI partners and who experience expanded autonomy in creative problem-solving exhibit heightened willingness to experiment with novel approaches. Building upon this theoretical logic, the following hypothesis is proposed.

H1: Human–machine collaboration directly and positively affects employees' digital creativity.

### 2.2 Mediating role of technology acceptance

One important theoretical framework for understanding people's acceptance and usage of information technology is the Technology Acceptance Model (TAM), proposed by Davis (1989). Perceived utility and ease of use are the two main components of this concept, and they influence a person's inclination to embrace technology and their usage habits (Davis, 1989). According to the reciprocal transformation mechanism in job demands–resources (JD-R) theory, different types of job resources (e.g., instrumental resources, psychological resources, and job engagement) can transform and reinforce one another (Xanthopoulou et al., 2009). Human–machine collaboration, as an instrumental resource, provides a practical foundation for enhancing employees‘ technology acceptance. When employees perceive the efficiency and convenience of intelligent systems in task execution, they become far more accepting and trusting of the technology. (Baroni et al., 2022). This heightened acceptance encourages employees to apply technological tools in innovative work contexts and experiment with new work methods to increase their digital creativity (Huu, 2023). Perceived usefulness in the Technology Acceptance Model is essential to the connection between human and machine collaboration and digital creativity. When employees believe that intelligent systems can effectively improve job performance, they are more inclined to explore the system's innovative potential and experiment with new features and applications (Wang et al., 2025). Such exploratory behavior, driven by perceived usefulness, effectively fosters employees' digital creativity. Similarly, usability in the technology acceptance model plays a pivotal role in human and machine collaboration and digital creativity. When employees find intelligent systems easy to use and understand, their cognitive load decreases, making them more willing to autonomously experiment with new functions and innovative applications (Ji et al., 2025). User-friendly system interfaces and interaction designs can reduce learning costs, allowing employees to focus more attention on creative tasks, thereby indirectly promoting innovative behavior. Notably, technology acceptance may have a dual effect when mediating the relationship between human and machine collaboration and digital creativity. Technology acceptance enhances digital creativity by encouraging employees to use and explore intelligent systems. On the other hand, excessive dependence on technological acceptance may have adverse effects. A study in the hospitality industry revealed that when employees perceive intelligent systems as “excessively useful,” it may lead to work complacency, weakening the positive impact of AI on creativity (Shi et al., 2025). This “too much of a good thing” phenomenon suggests a delicate balance between technology acceptance and innovation efficacy, where moderate skepticism and critical thinking may sometimes foster deeper innovation. Furthermore, the mediating role of technology acceptance is also influenced by the organizational innovation climate and technical support resources within the work environment (Stofberg et al., 2021). These moderating factors indicate that the mediating effect of technology acceptance is a complex process that must be understood within broader organizational and individual contexts. This study posits the following hypotheses by utilizing the aforementioned theoretical analysis and practical evidence.

H2: Technology acceptance plays a mediating role in human–machine collaboration and employee digital creativity.

### 2.3 The mediating effect of positive emotion

The role of positive emotions has become increasingly prominent in organizational behavior research. Fredrickson (2004) proposed that positive emotions can expand a person's thought–action repertoire“ and develop lasting personal assets, thereby fostering personal growth and development. Within the job demands-resources (JD-R) framework, positive emotions represent critical manifestations of the motivational process through which job resources translate into enhanced performance (Bakker and Demerouti, 2014; Edgar et al., 2018). A central theoretical question concerns why human-machine collaboration elicits predominantly positive emotions rather than the mixed reactions (e.g., anxiety about skill obsolescence, fear of job displacement) often observed during initial technology implementation. This study argues that net positive emotional responses emerge when employees appraise AI collaboration as a resource-providing rather than demand-imposing experience (Folkman et al., 1986; Bakker and Demerouti, 2017). Specifically, human-machine collaboration, conceptualized as complementary task allocation and mutual adjustment, functions as a socio-technical job resource that satisfies fundamental psychological needs through three mechanisms grounded in JD-R theory. First, competence satisfaction: effective collaboration enables employees to accomplish complex objectives that would be infeasible independently, generating feelings of mastery and self-efficacy that constitute core indicators of the motivational process (Gagné and Deci, 2005; Bakker and Demerouti, 2014). Second, resource accumulation via the gain spiral: acquiring AI capabilities initiates an upward cycle wherein resource accumulation begets positive emotional states, which in turn facilitate further creative engagement (Bakker and Van Wingerden, 2021). Third, demand reduction: by automating repetitive and cognitively demanding tasks, collaboration reduces job demands that deplete psychological resources, thereby preventing the negative affective states associated with strain and burnout (Demerouti et al., 2001). Critically, this study focuses on employees who have integrated AI into regular work routines, an implementation stage where the resource-providing nature of collaboration predominates over potential threat perceptions, resulting in predominantly positive emotional experiences.

In this study, positive emotions serve as the psychological mechanism linking human-machine collaboration with employees' digital creativity, grounded in the motivational process of JD-R theory (Bakker and Demerouti, 2017). By automating highly repetitive tasks, human-machine collaboration significantly reduces employees' workload, decreases psychological energy depletion (Demerouti et al., 2001), and releases psychological resources for emotional regeneration. These preserved resources alleviate fatigue and increase work engagement, thereby enhancing employees' cognitive flexibility, risk-taking willingness, and exploratory behavior (Shi et al., 2025). When employees experience positive emotions, characterized by pleasure, interest, and enthusiasm derived from effective AI collaboration, these affective states signal resource availability and activate the motivational process, broadening attentional scope and building the psychological resilience necessary for creative engagement (Fredrickson, 2004; Bakker and Demerouti, 2014). This motivational activation enables employees to break through traditional thinking patterns (Kiliç and Ercoşkun, 2024), discover novel connections, and generate innovative ideas. In the same way, positive emotions can broaden an individual's attention span, enhance cognitive flexibility, and promote associative thinking and remote associations, all of which facilitate creative idea generation (Kim, 2025). In light of these theoretical arguments, demonstrating that positive emotion (a) emerges from resource-provision appraisals in established collaboration contexts, (b) provides complementary explanatory power to technology acceptance through the affective-motivational pathway, and (c) activates the JD-R motivational process. This study proposes the following hypothesis.

H3: Positive emotions mediate the effect of human–machine collaboration on employees' digital creativity.

### 2.4 The chain mediating effect of technology acceptance and positive emotion

The chain mediation effect reflects the complex process of human–machine collaboration influencing employees' digital creativity, involving the continuous interaction of cognitive evaluation and emotional experience. The theoretical foundation of this hypothesis stems from the resource-gain spiral mechanism, in which initial resource acquisition facilitates further resource acquisition, forming a virtuous cycle of resource accumulation (Hobfoll, 2001). In this study, the chain mediation effect manifests as ”human–machine collaboration → technology acceptance → positive emotion → employee digital creativity.“ This pathway integrates instrumental cognition and emotional mechanisms, revealing the process of transformation from work resources to psychological resources. When employees exhibit greater technology acceptance, they are more willing to deeply utilize intelligent tools, thereby enhancing task completion quality and efficiency (instrumental resource gain), which further fosters a sense of achievement and control and triggers positive emotion (psychological resource gain). This emotional state, in turn, strengthens the willingness to explore new methods and cope with uncertainty, ultimately enhancing digital creativity (Mingjie et al., 2025). The transformation of technology acceptance into positive emotions aligns with cognitive appraisal theory. Employees view human–machine cooperation as an opportunity rather than a danger when they understand the value and simplicity of competence tools and are satisfied with their ability to utilize them, which results in positive emotional experiences (Paliga, 2022). Positive emotional states not only broaden cognitive scope but also enhance cognitive flexibility, fostering exploratory thinking and risk-taking behaviors. Employees are more inclined to attempt new strategies, form new relationships, and overcome mental preconceptions when they are in a favorable emotional state. The formation of a chain mediation effect also requires support from organizational environments and individual factors, such as the organizational innovation climate, leadership support, colleague relationships, individual learning goal orientation, growth mindset, and psychological capital. These factors can further facilitate the transformation of technology acceptance into positive emotions, ensuring the sustained operation of the resource gain spiral. In summary, the selection of positive emotion alongside technology acceptance reflects the cognitive-affective dual-process framework within JD-R theory (Bakker and Demerouti, 2014). While alternative mediators such as psychological safety or creative self-efficacy are theoretically plausible, technology acceptance and positive emotion offer distinct yet complementary explanatory mechanisms that together provide a more complete account of how human-machine collaboration enhances creativity. Technology acceptance represents the cognitive-utilitarian pathway, capturing how employees leverage AI resources through perceptions of usefulness and ease of use (Davis, 1989). This pathway addresses the instrumental capacity to utilize AI tools effectively. In contrast, positive emotion constitutes the affective-motivational pathway, capturing why employees are willing to deploy these resources for creative exploration, tolerate ambiguity, and persist through innovation failures. While cognitive acceptance explains the ability to work with AI, positive emotion explains the willingness to experiment creatively with AI—a distinction crucial for understanding digital creativity, which requires both capability and motivational readiness (Zhou and Hoever, 2014). This dual-pathway configuration aligns with JD-R theory's proposition that job resources simultaneously activate motivational processes through multiple channels (Schaufeli, 2015). This leads to the following hypothesis being put forth in this study.

H4: Technology acceptance and positive emotions jointly play a chain-mediating role in human–machine collaboration and employee digital creativity.

### 2.5 Research model

**Figure 1:**
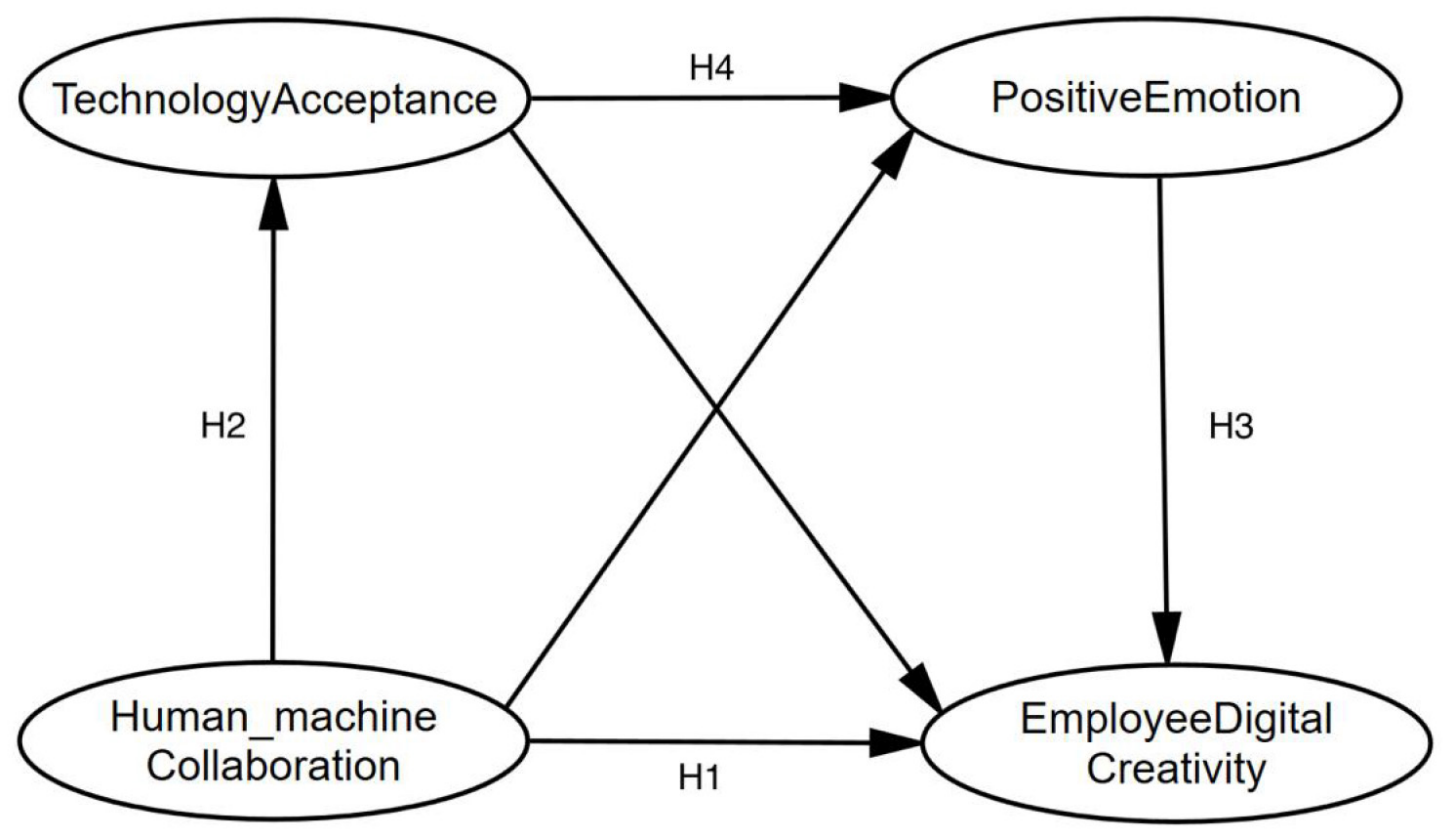
Research hypothesis model.

Building on the research hypotheses proposed above, this study develops a theoretical model of how employees' digital creativity is affected by human–machine collaboration, as illustrated in Figure 1. In keeping with the principle of job demands–resources (JD-R), the model positions human–machine collaboration as the independent variable, incorporates technology acceptance and positive emotion as mediating variables, and designates employees' digital creativity as the dependent variable. To test this model, structural equation modeling (SEM) was employed as the data analysis approach, and SPSS and AMOS software were used for model fitting and validation. By examining this theoretical framework, this study aims to move beyond the simplistic verification of direct effects, delving into the intrinsic dynamics through which human–machine collaboration empowers employees' digital creativity. The findings are intended to provide enterprises with theoretical guidance and practical insights for effectively managing human–technology interactions and maximizing innovation output in the digital era.

## 3 Research method

### 3.1 Variable measurement

To ensure data quality, the scales used in this study were referenced from those validated in prior research with good reliability and validity. The measurement employed a 5-point Likert scale, where “1” indicates “strongly disagree” and “5” represents “strongly agree.”

#### 3.1.1 Human–machine collaboration

This scale's assessment revolves around MEDCOF J W's Computer Program Utilization Scale (Medcof, 1996), with adaptations based on interviews with employees from digital-intelligent enterprises to identify context-specific items. The adaptation process involved three stages: First, interviews were conducted with employees to understand their actual collaboration experiences with AI systems. Second, experts in organizational psychology and AI research reviewed the items for content validity. Third, a pilot test was conducted to assess item clarity and reliability, resulting in the final four-item scale. It includes four items, such as “AI technology provides information resources to assist me in completing my work” and “I interact with AI technology in real-time to improve work efficiency.”

#### 3.1.2 Technology acceptance

This scale's measurement draws on the Technology Acceptance Scale developed by Davis and Malik Sallam (Davis, 1989), which has six overall items and two dimensions: perceived utility and simplicity of application. Examples include “Generative AI tools help me save time when searching for information” and “I can quickly learn how to use generative AI tools.”

#### 3.1.3 Positive emotions

This rating system was measured via the Kim et al. (2018) scale, which includes positive emotions such as happiness, enthusiasm, confidence, and focus. It consists of four items, with the following instructions: “Please indicate to what extent you have experienced the following emotions during your work with AI systems in the past week.”

#### 3.1.4 Employees' digital creativity

The measurement of this scale adopts the scale proposed by BAER et al. (Baer and Oldham, 2006), which consists of four items and is unidirectionally assessed by employees' direct supervisors, such as ”the level of activity in proposing new ideas for improving work plans.“

### 3.2 Sample data collection

This study employed a cross-sectional multi-source survey design. Employees from digital and intelligent businesses in various industries, including intelligent manufacturing, intelligent transportation, intelligent medicine, and new sales, were the main target audience for this study's questionnaire-based data collection. The enterprises were located primarily in four regions: Shanghai, Zhejiang, Anhui, and Jiangsu. The survey was conducted online and offline. The online surveys utilized a professional online questionnaire platform employing maximum variation sampling to select employees from digital and intelligent enterprises. Offline questionnaires were collected mainly through purposive sampling involving visits to enterprises that had adopted and used AI technology systems, where questionnaires were uniformly sent to their employees. A total of 473 surveys were conducted. After eight duplicate samples were removed on the basis of user mobile numbers and 64 samples with identical answers to most questions were excluded, 401 valid questionnaires were obtained. Table 1 presents the statistical analysis results. Among the respondents, 50.87% and 49.13% were male and female, respectively, indicating a relatively balanced gender distribution, with slightly more male respondents. More than 80% of the respondents had a bachelor's degree or higher. The roles covered include sales, finance, human resources or administration, and product production or service provision, with a relatively even distribution across these fields. This suggests that the study sample has a solid foundation in terms of employees' digital creativity and is reasonably diverse but non-random.

**Table 1:** Descriptive statistics of survey samples.

Basic Classification	Indicator	Frequency	Frequency%
Gender	Male	204	50.87
	Female	197	49.13
Age	18–30 years old(inclusive)	101	25.19
	30–45 years old(inclusive)	256	63.84
	Over 45 years old	44	10.97
Educational attainment	Junior college or below	79	19.70
	Bachelor's degree	214	53.37
	Master's degree and doctoral degree	108	26.93
Seniority	3 years or less	126	31.42
	3–10 years(inclusive)	211	52.62
	More than 10 years	64	15.96
Company size	Fewer than 50 people	83	20.70
	50–300 people(inclusive)	122	30.42
	300–500 people(inclusive)	101	25.19
	More than 500 people	95	23.69
Company age	5 years or less	94	23.44
	5–10 years(inclusive)	296	73.82
	More than 10 years	11	2.74
Position	Sales	97	24.19
	Finance	106	26.43
	Human resources or administrative management	84	20.95
	Product production or service provision	114	28.43

### 3.3 Data analysis

Prior to conducting empirical tests, this study utilized SPSS 27.0 and AMOS 24.0 for data processing, establishing a multidimensional and systematic analytical framework centered on data quality assessment. This framework encompasses essential phases, such as typical technique bias assessment, reliability evaluation, and integrity review, establishing the data basis for further computational equation modeling and ensuring the scientific rigor and dependability of the study outcomes. Common method bias refers to the artificial inflation of spurious correlations among variables because all variables are measured via the same instrument, constituting a type of systematic measurement error common in self-report questionnaire studies (Podsakoff et al., 2003). In this research, three core variables—“human–machine collaboration,” “technology acceptance,” and “positive emotion”—were measured via employee self-evaluations, whereas “employee digital creativity” employee digital creativity was assessed by direct supervisors. Therefore, potential common method bias requires rigorous examination. This study used Harman's single-factor test to identify common technique bias. An unrotated exploratory factor analysis revealed a dominant factor with a variance explanation rate significantly higher than that of other factors, normally above 40%, indicating the presence of severe common method bias in the data according to the fundamental logic of this method (Zhonglin, 2020). The test results revealed two dominant factors with eigenvalues greater than one, with the first factor explaining 18.904% of the variance. According to the criteria for common method bias, this indicated the absence of severe bias and no single dominant factor, confirming good data independence and suitability for further analysis.

The main factors were measured via a scale. Therefore, conducting reliability tests on measurement data is a prerequisite to ensure the practical significance of subsequent analyses. This study employed the Cronbach's alpha coefficient as the key metric for reliability testing. This metric primarily reflects homogeneity among items within the scale, with a value range of 0–1. In general, a scale's internal consistency reliability is high when its Cronbach's α coefficient is above 0.8; it is adequate when it is between 0.7 and 0.8; if it is below 0.7, scale item modifications or deletions are necessary. Table 2 presents the experimental outcomes. The four questions on the “Human–Machine Collaboration” scale have a Cronbach's α coefficient of 0.918, which reflects a high level of internal consistency and consistent measurement of employees‘ real experiences with human–machine cooperation. With six items and a Cronbach's α value of 0.923, the “Technology Acceptance” measure exhibited good internal consistency in evaluating perceived utility as well as simple use, accurately capturing employees' cognitive assessments of AI technology. The “positive emotion” scale contains four items, with a Cronbach's α coefficient of 0.895, indicating that the scale stably reflects employees‘ positive emotional states over the past 3 months, with high homogeneity among the items. The “Employee Digital Creativity” scale comprises four items, with a Cronbach's α coefficient of 0.908, illustrating consistent evaluation criteria by leaders for employees' creative behaviors and stable, reliable measurement results from the scale.

**Table 2:** Reliability analysis.

Factor	Cronbach's α	Number of projects
Human–machine collaboration	0.918	4
Technology acceptance	0.923	6
Positive emotion	0.895	4
Employee digital creativity	0.908	4

To ascertain whether the scale items could be suitably categorized under their intended constructions, this study initially used exploratory factor analysis (EFA) to assess the scale's construct validity prior to conducting confirmatory factor analysis. The principal component approach was used by the EFA to identify common factors, and variable rotation was used to clarify the relationships between factors and items. The Kaiser–Meyer–Olkin (KMO) measure of sample adequacy and Bartlett's test of sphericity are commonly used to evaluate appropriateness. The KMO value, which ranges from 0 to 1, quantifies the partial correlation between the variables. The data are extremely suitable for factor analysis when the KMO value is greater than 0.8; values between 0.7 and 0.8 are deemed adequate; values between 0.6 and 0.7 are moderately acceptable; and values less than 0.6 are inappropriate. Using Bartlett's test of sphericity, one can determine whether the correlation matrix between variables is an identity matrix. The data are suitable for factor analysis if the test result is significant (p < 0.05), which indicates substantial correlations between the variables (Kaiser, 1974). The KMO value, as displayed in Table 3, is 0.947, which is well within the permissible range of above 0.8. The corresponding significance probability (Sig) was 0.000, which was below the threshold of 0.05. The questionnaire structure was valid, with strong correlations between variables, confirming the overall suitability of the data for confirmatory factor analysis.

**Table 3:** KMO and bartlett's test.

Indicator	Value
Kaiser-Meyer-Olkin measure of sampling adequacy	0.947
Bartlett's test of sphericity approximate chi-square	6,561.2
Df	153
Sig.	0.000

**Figure 2:**
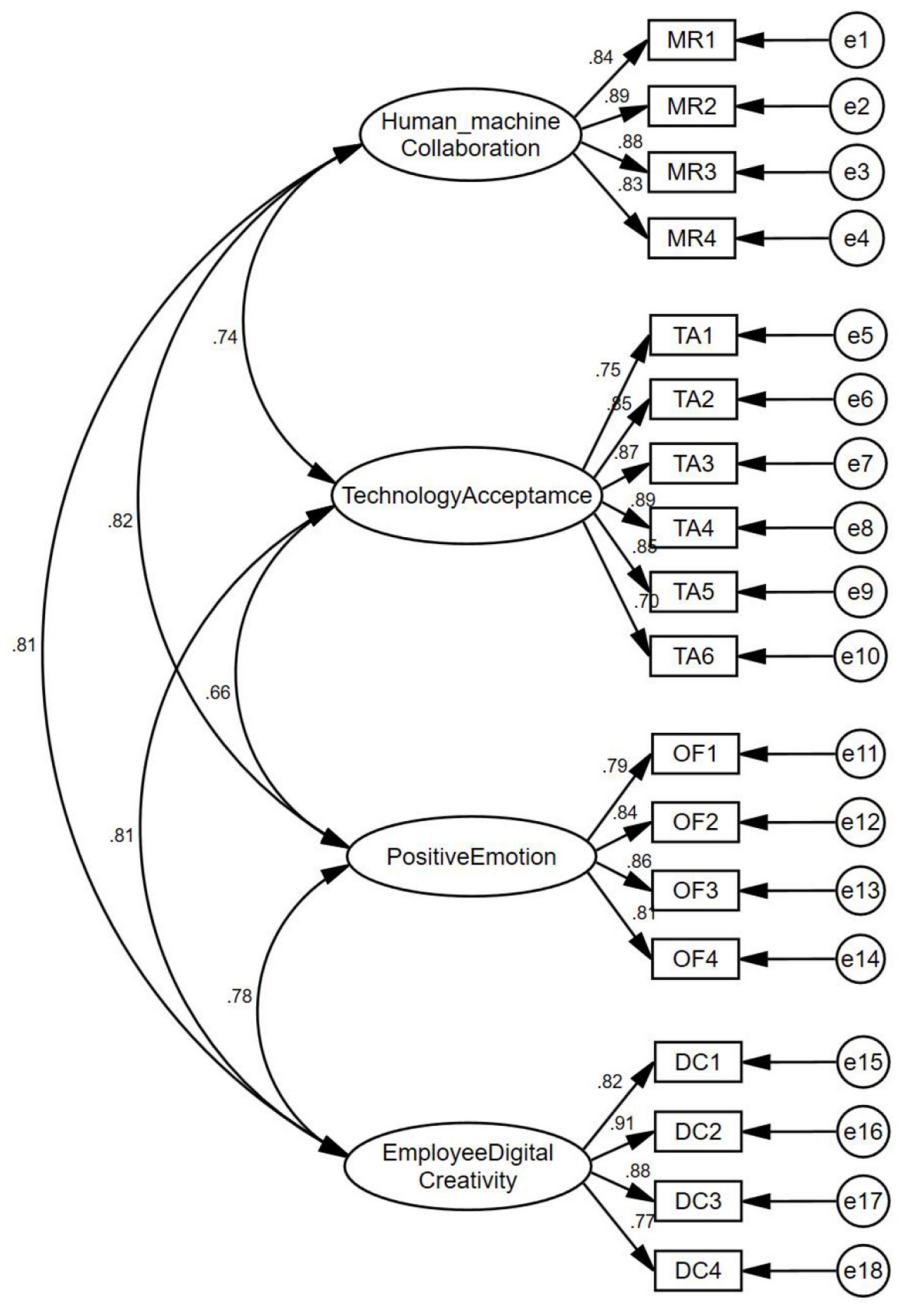
Confirmatory factor analysis (CFA) model.

Confirmatory factor analysis (CFA) builds upon exploratory factor analysis (EFA) by constructing structural equation models to validate predefined factor structures, thereby examining the convergent and discriminant validity of scales more precisely. In this study, AMOS software (version 24.0) was used to perform CFA on the four core variables, with the constructed CFA model shown in Figure 2. The experimental results are presented in Table 4. All the items demonstrated excellent standardized factor loadings: the four items for human–machine collaboration had standardized factor loadings between 0.830 and 0.890, well above the 0.5 threshold, indicating significant relationships between the items and the construct; the six items for technology acceptance had standardized factor loadings ranging from 0.704 to 0.887, with all the items' C.R. values greater than 14.48, p < 0.001, indicating that the perceived usefulness and perceived ease of use items effectively measure the technology acceptance construct; the standardized factor loadings for the four positive emotion items fluctuated between 0.788 and 0.860, all of which were over 0.5, suggesting that the measures consistently reflect positive affective states; and the four items for employee digital creativity had standardized factor loadings varying from 0.773 to 0.911, indicating that the supervisor-rated items effectively measure employees' digital creativity.

**Table 4:** Confirmatory factor analysis results.

Variable	Title	Nonstandardized factor loadings	S.E.	C.R.	*P*	Standardized factor loadings	AVE	CR
Human–machine collaboration	MR1	1				0.842	0.740	0.919
	MR2	1.078	0.047	23.04	^***^	0.89		
	MR3	1.042	0.046	22.503	^***^	0.878		
	MR4	0.99	0.048	20.53	^***^	0.83		
Technology acceptance	TA1	1				0.754	0.676	0.926
	TA2	1.141	0.064	17.966	^***^	0.848		
	TA3	1.308	0.07	18.621	^***^	0.875		
	TA4	1.426	0.075	18.93	^***^	0.887		
	TA5	1.312	0.073	17.959	^***^	0.848		
	TA6	1.036	0.072	14.484	^***^	0.704		
Positive emotion	OF1	1				0.788	0.682	0.895
	OF2	1.087	0.059	18.584	^***^	0.845		
	OF3	1.068	0.056	18.992	^***^	0.86		
	OF4	1.013	0.058	17.561	^***^	0.808		
Employee digital creativity	DC1	1				0.823	0.721	0.912
	DC2	1.218	0.053	22.905	^***^	0.911		
	DC3	1.138	0.052	21.841	^***^	0.883		
	DC4	0.992	0.055	17.892	^***^	0.773		

^***^ indicates *P* < 0.001.

Discriminant validity refers to the significant differences between different constructs, meaning that the correlation between one construct and others should be lower than that with its own measurement items. This study employs the Fornell–Larcker criterion to assess the legitimacy of discrimination. The core judgment standard of this criterion is that, for any two factors A and B, the standardized correlation coefficient between factors A and B should be smaller than the AVE value of factor A squared (Ab Hamid et al., 2017). First, the standardized correlation coefficients among the four factors were calculated, and the results are presented in Table 5. As Table 5 indicates, the correlation coefficients between the constructs all fall within a reasonable range: the correlation coefficient between human–machine collaboration and technology acceptance is 0.738, suggesting that the higher the degree of employee participation in human–machine collaboration is, the greater the degree of acceptance of AI technology typically is; the correlation coefficient between technology acceptance and positive emotion is 0.818, suggesting that human–machine collaboration can successfully lessen workers‘ workloads and improve their pleasant emotional experiences; the correlation coefficient between human–machine collaboration and employee digital creativity is 0.806, providing preliminary evidence for the beneficial effects of human–machine collaboration on digital creativity; the correlation coefficient between technology acceptance and positive emotion is 0.657, suggesting that employees' cognitive recognition of AI technology (technology acceptance) may positively influence their emotional state; and the correlations between technology acceptance and employee digital creativity (0.806) and between positive emotion and employee digital creativity (0.782) are both significantly positive, offering initial support for subsequent mediation effect testing. The square roots of the AVE values for each aspect were calculated. For example, the square root of the AVE for employee digital creativity was 0.849, that for technology acceptance was 0.822, that for positive emotion was 0.826, and that for human–machine collaboration was 0.860. According to the comparative analysis, the standardized coefficients of association between each pair of items in this discriminant validity test are all less than the corresponding AVE square roots. Thus, the study demonstrated good discriminant validity among the factors, with no construct confusion issues. This indicates that each scale independently and accurately measures the corresponding research factors.

**Table 5:** Results of the discriminant validity test for each dimension.

Variable	Human–machine collaboration	Technology acceptance	Positive emotion	Employee digital creativity
Human–machine collaboration	**0.74**			
Technology acceptance	0.738	**0.676**		
Positive emotion	0.818	0.657	**0.682**	
Employee digital creativity	0.806	0.806	0.782	**0.721**
Square root of AVE value	0.860	0.822	0.826	0.849

Bold values on the diagonal represent the square root of the average variance extracted (AVE) for each construct.

### 3.4 Data results

**Figure 3:**
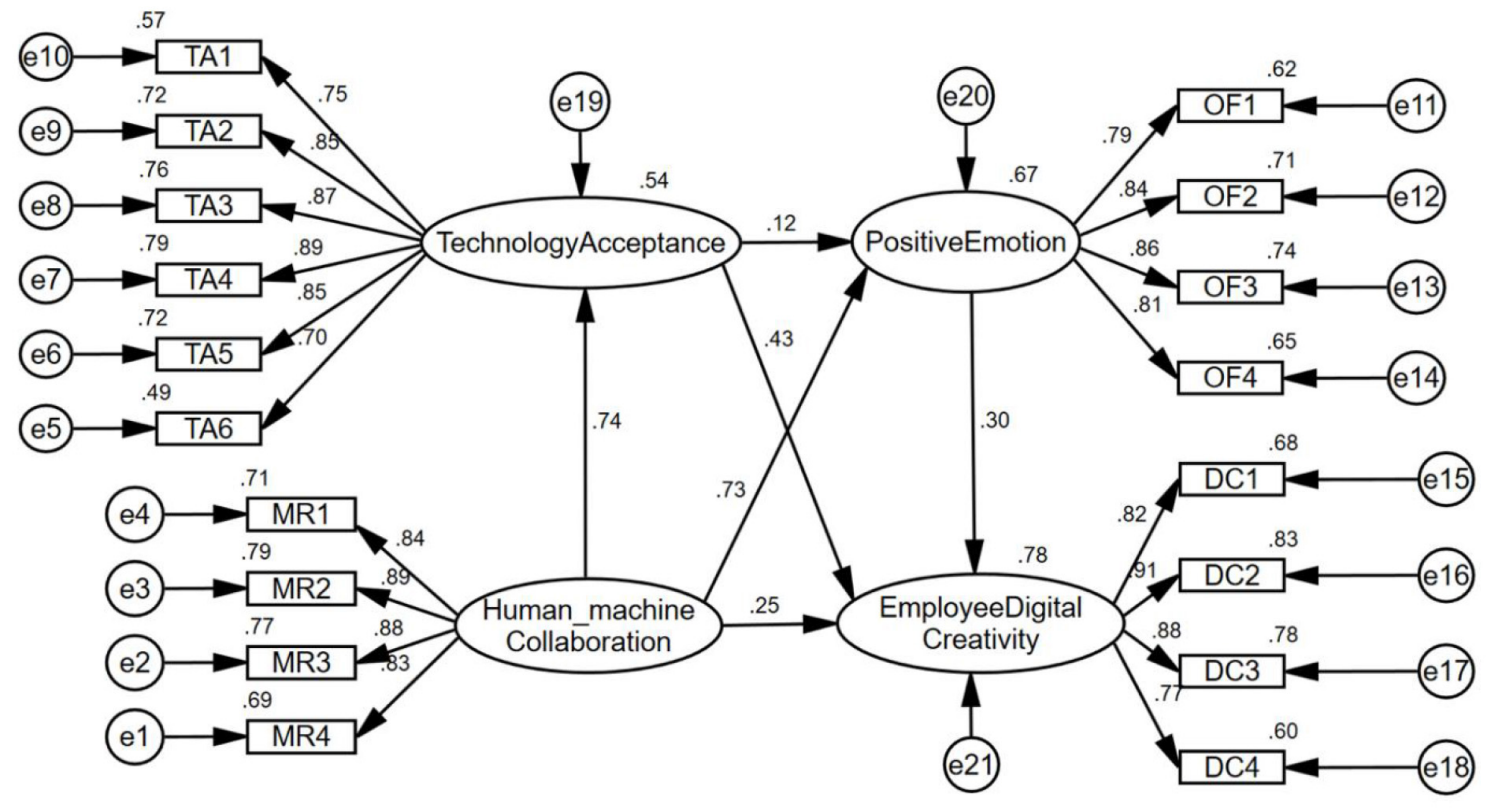
Standardized structural equation model.

This study employed AMOS software to construct a structural equation model incorporating four core variables—human–machine collaboration, technology acceptance, positive emotion, and employee digital creativity—and validated the relationships among these variables. The final structural equation framework is shown in Figure 3 after modifications were made on the basis of the conceptual model proposed in this study and the data gathered from the questionnaire.

The goodness-of-fit of the structural equation model is a key indicator for evaluating the synchronization of the sample data and the model, directly affecting the reliability of the path coefficients and mediation effect test results. This study refers to commonly used fit indices in organizational behavior and psychological research to assess model fit from three dimensions: absolute fit, relative fit, and parsimonious fit. Taking into account the model fit test results of Table 6, a chi-square degree of freedom ratio (CMIN/DF) below three generally indicates good model fit, whereas a value between 1 and 2 suggests an excellent fit. In this study, with CMIN=687.251 and DF=329, the calculated CMIN/DF was 2.089, falling between 1 and 3 and approaching the ideal benchmark of 2. This demonstrates that the model fits the data well, with acceptable discrepancies between the predicted and observed values. The root mean square error of approximation (RMSEA) measures the discrepancy between a theoretical model and observed data, taking into account the complexity of the model, and is one of the core metrics for evaluating the fit of structural equation models. Excellent model fit is indicated by an RMSEA below 0.05, medium fitness is given by an RMSEA between 0.05 and 0.08, marginal fitting is suggested by an RMSEA between 0.08 and 0.10, and values exceeding 0.10 signify poor fit. In this study, the RMSEA of 0.034 was well below the threshold of 0.05, demonstrating minimal approximation error and exceptional alignment with the sample data. Relative fit indices assess model improvement by contrasting the study framework with a “baseline model,” with key metrics including the GFI, AGFI, CFI, NFI, and TLI. In this study, the GFI of 0.933 and AGFI of 0.919 both exceeded the critical threshold of 0.9, indicating that the model effectively explained the variance and covariance of the observed variables. Furthermore, the comparative fit index (CFI) and normed fit index (NFI) significantly surpassed the 0.9 benchmark, meeting all the fit criteria and further validating the model's robustness.

**Table 6:** Model fit indices.

Indicator	CMIN	DF	CMIN/DF	RMSEA	GFI	AGFI	CFI	NFI	TLI
Acceptable range			< 3	< 0.05	>0.9	>0.9	>0.9	>0.9	>0.9
Measured value	687.251	329	2.089	0.034	0.933	0.919	0.914	0.917	0.908

Under the premise of good model fit, this study examines the direct influence of human–machine collaboration on employee digital creativity and the mediating path effects of technology acceptance and positive emotion between them through a path coefficient test of the structural equation framework. The path coefficient (estimate) directly reflects the strength of the impact of the connection between variables, whereas the path coefficient's importance is tested via the critical ratio (C.R.), with particular findings displayed in Table 7. According to research hypothesis H1 and the table data, the path coefficient from human–machine collaboration to employees‘ digital creativity is 0.245, with a significance level of *p* < 0.001 (denoted as “^***^” in the table). The path coefficient is significantly positive, indicating that H1 is fully validated. Research hypothesis H2 consists of two mediating subpaths: “human-machine collaboration → technology acceptance” and “technology acceptance → employee digital creativity.” According to Table 7, the path coefficient between human and AI collaboration and technology acceptance is 0.738, indicating that the more employees engage in human-machine collaboration, the greater their acceptance of AI technology. The path coefficient between technology acceptance and employee digital creativity was 0.428, suggesting that greater acceptance of AI technology leads to stronger digital creativity among employees. Combining the results of these two subpaths, the mediating role of technology acceptance linking human-machine collaboration to employee digital creativity is statistically significant, confirming Hypothesis H2. According to H3, which also includes two subpaths, the path coefficient from human–machine collaboration to positive emotion is 0.731, with a CR value of 10.543 (greater than 3.29) and p < 0.001, indicating a significantly positive path coefficient. This finding demonstrates that human–machine collaboration can significantly enhance employees' positive affective experiences, aligning with the “psychological resource regeneration mechanism” of JD-R theory (Bakker and Demerouti, 2017). The path coefficient from positive emotion to employees‘ digital creativity was 0.300, showing that stronger positive affective experiences lead to higher levels of digital creativity. Combining the results of both subpaths, the significance of good affect as a mediator connecting employee digital creativity with human–machine collaboration is validated, thus supporting H3. Research hypothesis H4 posits that technology acceptance and positive emotions play a chain-mediating role between human–machine collaboration and employees' digital creativity. This chain path consists of three subpaths: “human–machine collaboration → technology acceptance,” “technology acceptance → positive emotions,” and “positive emotions → employees‘ digital creativity.” As shown in Table 7, the path coefficient for “human–machine collaboration → technology acceptance” has been validated as 0.738 (p < 0.001), whereas the path coefficient for “positive emotions → employees' digital creativity” has been validated as 0.300 (p < 0.001). The critical subpath “technology acceptance → positive emotions” has a path coefficient of 0.117, with a significance level of *p* = 0.019 (less than 0.05). The positive path coefficient indicates that technology acceptance has certain beneficial effects on positive emotions, supporting H4 in the early stages.

**Table 7:** Structural equation model path coefficients.

Path	Estimate	S.E.	C.R.	*P*	Significance
Technology acceptance < - human–machine collaboration	0.738	0.055	12.330	^***^	Significant
Positive emotion < - technology acceptance	0.117	0.062	1.968	^*^	Significant
Positive emotion < - human–machine collaboration	0.731	0.066	10.543	^***^	Significant
Employee digital creativity < - positive emotion	0.300	0.068	4.509	^***^	Significant
Employee digital creativity < - technology acceptance	0.428	0.058	7.898	^***^	Significant
Employee digital creativity < - human–machine collaboration	0.245	0.071	3.416	^***^	Significant

^***^ indicates *P* < 0.001; ^*^ indicates *P* < 0.05

To examine the underlying mechanisms through which human–machine collaboration influences employee digital creativity, this study tests three mediating paths using the bootstrap method with 5,000 resamples (Zhang et al., 2010). This approach provides superior statistical power and more accurate bias-corrected confidence intervals compared to traditional Sobel tests. Table 8 summarizes the empirical results. The test results show that the mediating effect value for the “human-machine collaboration → technology acceptance → employee digital creativity”, the indiect effect is 0.310 (BootSE = 0.056). The 95% bias-corrected confidence interval [0.200, 0.426] excludes zero, confirming a significant mediation effect and supporting Hypothesis H2. For the second independent mediation path “human-machine collaboration → positive emotion → employee digital creativity”, the indirect effect is 0.216 (BootSE = 0.067). The 95% confidence interval [0.096, 0.362] excludes zero, verifying a significant mediating role and validating Hypothesis H3. The serial mediation chain “human-machine collaboration → technology acceptance → positive emotion → employee digital creativity”, the indirect effect is 0.025 (BootSE = 0.018). The 95% confidence interval [0.007, 0.073] excludes zero, demonstrating a significant serial mediation effect and supporting Hypothesis H4. Collectively, the three indirect pathways yield a total mediation effect of 0.552 (BootSE = 0.088, 95% CI [0.399, 0.713]), explaining 69.7% of the total effect of human–machine collaboration on employee digital creativity.

**Table 8:** Results of the mediation effect test.

Path	Mediation effect value	BootSE	BootLLCI	BootULCI
Human–machine collaboration—technology acceptance—employee digital creativity	0.310	0.056	0.200	0.426
Human–machine collaboration—positive emotion—employee digital creativity	0.216	0.067	0.096	0.362
Human–machine collaboration—technology acceptance—positive emotion—employee digital creativity	0.025	0.018	0.007	0.073
Total mediating effect	0.552	0.088	0.399	0.713
Total effect	0.792	0.053	0.682	0.896

## 4 Conclusions and discussion

### 4.1 Research conclusions

This study examines associations among 401 valid questionnaire responses, employs structural equation modeling (SEM) to explore the theoretical model and investigate potential pathways through which human–machine collaboration relates to employees' digital creativity via technology acceptance and positive emotions. The main findings are as follows.

First, employees' digital inventiveness has been substantially increased by human–machine collaboration. This finding validates Hypothesis H1, indicating that in the current era where AI technology is deeply integrated into workflows, human–machine collaboration may function as a critical work resource rather than merely ”tool assistance“. Such collaboration appears to help employees reduce repetitive tasks and improve efficiency through AI capabilities, while also providing diverse perspectives and technical support during the innovation process. This pattern is consistent with the ”resource gain principle“ of job demands-resources theory (Bakker and Demerouti, 2014). When workers report effectively collaborate with AI in practical work, such as using AI for foundational tasks, such as data filtering and model construction, they are more likely to perceive AI as a partner that supports innovation rather than just a tool. This shift in perception further stimulates their willingness to explore new methods and experiments with creative ideas, ultimately directly enhancing digital creativity.

Second, technology acceptance appears to mediate the relationship between human–machine collaboration and employees' digital creativity. The indirect effect value of technology acceptance is 0.2863 (95% CI = [0.2089, 0.3656]), suggesting that technology acceptance may serve as a cognitive pathway through which human–machine collaboration relates to creativity, consistent with Hypothesis H2. Technology acceptance primarily manifests in employees' perceptions of AI tools' usefulness ”usefulness“ and ”ease of use“, where ”usefulness“ refers to employees believing that AI tools can help them better achieve work goals, and ”ease of use“ refers to employees perceiving low difficulty in mastering and using AI tools. In human–machine collaboration scenarios, employees who report perceiving the usefulness of AI tools—such as recognizing AI's ability to quickly generate data analysis reports or optimize workflows—while also finding AI interfaces intuitive and learning costs low, tend to explore AI functionalities and apply them to solving complex problems. This behavioral process aligns with the logical framework of the technology acceptance model, emphasizing cognitive factors may function as important antecedents of individual behavioral change. In this study, employees' positive perceptions of AI technology bridge human–machine collaboration and digital creativity, potentially enabling the resource advantages of collaboration to connect with tangible improvements in employees' digital creativity through cognitive transformation.

Third, human–machine collaboration shows an indirect association with employees' digital creativity through positive emotions. In traditional work models, employees often experience ”innovation fatigue“ due to high task pressure and excessive psychological energy depletion, leading to fatigue, irritability, and diminished innovative behavior. However, in a human–machine collaborative environment, AI tools may help employees reduce task pressure by handling high-load, repetitive tasks such as document organization, allowing them to focus more on core creative tasks such as ideation. This allows them to focus more on core creative tasks such as ideation. The alleviation of workload fosters more positive emotional experiences; for instance, when employees use AI to quickly complete time-consuming tasks, they gain a sense of achievement from improved efficiency. These positive emotions are linked to innovative thinking and initiative, expanding employees' cognitive boundaries and exploratory behaviors, thereby providing psychological momentum for creative activities (Zhou and Hoever, 2014). Thus, positive emotions may not only enhance immediate work experiences but also subtly offer a sustained psychological drive for creative endeavors, supporting employees' engagement in digital innovation practices.

Fourth, a chain mediation effect exists between technology acceptance and positive emotion. From a psychological mechanism perspective, employees‘ perception of human–machine collaboration begins at the cognitive level. When exposed to AI tools, employees first assess their usefulness and ease of use, resulting in a cognitive outcome of technology acceptance. According to Lazarus (1991) cognitive appraisal theory, individuals‘ emotional experiences are influenced by cognitive evaluations. Employees who report cognitively recognizing the benefits and ease of AI technology tend to exhibit positive emotional experiences at the affective level, such as favorable attitudes toward AI tools and feelings of enjoyment during use. This positive emotion is then associated with innovative behaviors, potentially motivating employees to proactively apply AI tools in digital creative practices. This chain mediation pattern is consistent with the psychological sequence of “cognition → affect → behavior” and offers a nuanced perspective for understanding employees' innovation psychology in human–machine collaboration contexts.

### 4.2 Theoretical contributions

This study advances theory in three significant ways by identifying conceptual gaps in existing literature and providing nuanced extensions., and grounded in the Job Demands-Resources (JD-R) theory, reveals the complex pathways through which human–machine collaboration influences digital creativity. The theoretical model integrates JD-R theory, the technology acceptance model, and the positive impact pathway, forming a comprehensive framework to explain how human–machine collaboration affects employees' digital creativity. The value of this theoretical integration lies in its revelation of multilevel mechanisms, which consider both the rational pathway of tool cognition (technology acceptance) and the emotional pathway of affective experience (positive emotion), while also examining their chained mediation effects. This multidimensional perspective provides comprehensive theoretical guidance for understanding human–machine collaboration in today's digital work environments, particularly as digital transformation comprehensively impacts value creation and innovation paradigms across industries (Vaska et al., 2021). From an empirical standpoint, the chained mediation model validated in this study is well supported by the data (indirect effect = 0.0372, 95% CI = [0.0121, 0.0724]). This finding aligns with the perspective proposed by Bandura (1986), which emphasizes the interplay between cognition, behavior, and affect, suggesting that an individual's use and evaluation of technology can influence their emotional state, thereby modulating behavioral performance. Muneer et al. (2024) also highlighted the mediating role of psychological empowerment in digital transformation and innovative behavior, proposing that psychological empowerment serves as a mediator that positively links innovative behavior with digital transformation.

First, the study expands the connotation of the job demands-resources (JD-R) theory by identifying human–machine collaboration as a new type of job resource. Since its inception, the definition of “job resources” in JD-R theory has continuously evolved with social development and changing work dynamics, progressing from traditional resources, such as social support and job autonomy, to technological resources in digital contexts (Bakker and Demerouti, 2024). A critical conceptual gap remains: existing research conceptualizes technological resources primarily as static tools or equipment that employees possess and use, overlooking the interactive, partnership-based nature of human-machine collaboration. Prior studies examining digital work resources (Zia et al., 2024) have predominantly focused on the direct effects of such resources on task performance or innovative work behavior, treating technology as a unidirectional input that directly enhances outcomes through instrumental utility. This study extends JD-R theory by reconceptualizing human-machine collaboration not as a static “digital tool” but as a “compound job resource” characterized by dynamic bidirectional interaction between human intelligence and artificial intelligence. Specifically, study extend the traditional “resource → outcome” direct effect model—which has dominated JD-R research—by revealing dual sequential transformation mechanisms: (1) the instrumental transformation pathway, wherein technology acceptance serves as a cognitive bridge that translates external technological capabilities into internalized resource perceptions; and (2) the psychological regeneration pathway, wherein positive emotion functions as an affective mechanism that accumulates psychological capital from collaborative experiences. This extension addresses a critical limitation in prior JD-R research: while studies have acknowledged that resources can promote “personal growth and development” (Hlado and Harvankova, 2024), they have rarely specified how technological resources regenerate psychological resources through emotional pathways. By demonstrating that human-machine collaboration enhances creativity not merely through direct instrumental support (AI computing power, data processing capabilities) but through the sequential cultivation of cognitive acceptance and positive emotion, this study reveals that digital-era job resources operate through multi-stage psychological processing rather than simple direct transmission. The validated chain mediation (indirect effect = 0.0372, 95% CI = [0.0121, 0.0724]) provides empirical evidence that JD-R theory must account for cognitive-affective sequences when explaining how novel technological resources influence creative outcomes in AI-enabled workplaces. While prior studies have focused predominantly on the direct impact of technological resources on task performance, this study delves deeper into the psychological dimension, uncovering how technological resources indirectly influence innovative behavior through emotional pathways.

Second, the interaction mechanism between cognition and affect was validated, thus enriching the affective dimension of the Technology Acceptance Model (TAM). The Technology Acceptance Model (TAM) and its extensions (TAM2, TAM3, UTAUT, UTAUT2) have established a robust paradigm for understanding technology adoption, emphasizing cognitive rationality—perceived usefulness and perceived ease of use—as primary determinants of usage intentions and behaviors (Davis, 1989). While recent extensions have incorporated hedonic motivation and social influence (Venkatesh et al., 2012), a fundamental theoretical gap persists: these models treat cognitive and affective mechanisms as parallel or competing predictors rather than sequential processes, and they fail to specify how cognitive evaluation of technology translates into emotional experiences that drive complex behavioral outcomes like creativity. This study extends TAM by establishing technology acceptance as an antecedent to positive emotion, thereby revealing a cognitive-to-affective progression that traditional models have not adequately conceptualized. Unlike prior research that examines technology acceptance and emotional responses as separate mediators (Hsu and Lin, 2022), our serial mediation findings suggest that cognitive recognition of technology value precedes and cultivates emotional responses, which subsequently fuel creative behavior. This represents a significant theoretical advance: rather than viewing cognition and affect as alternative pathways (the “which matters more” question), we demonstrate that they operate as complementary stages in a psychological process where cognitive assessment creates the foundation for emotional engagement. Furthermore, this study extends TAM's applicability beyond traditional “adoption intentions” and “usage behaviors” to “creative performance”—a complex, emotion-dependent outcome that requires not merely accepting technology but experiencing it as empowering. By specifying the emotional pathway (positive emotion) through which cognitive technology evaluation (technology acceptance) translates into innovative outcomes (digital creativity), we address a critical void in technology acceptance literature: how and why accepted technology generates creative benefits. This aligns with and extends Venkatesh et al. (2012) UTAUT2 findings on hedonic motivation by providing a mechanistic explanation of the cognitive-affective-creative sequence, rather than simply documenting that both cognition and emotion matter. This underscores the fundamental role of psychological mechanisms as active mediators that can boost creative outcomes, a dynamic similarly supported by research demonstrating how internal psychological beliefs—such as creative efficacy and trust—sequentially mediate and enhance group-level creativity (Fan et al., 2021).

Finally, the study deepened the exploration of the factors influencing digital creativity and expanded interdisciplinary theoretical dialogue. While prior research on digital creativity has examined individual factors (e.g., digital skills, creative self-efficacy) and contextual factors (e.g., leadership, organizational climate) in isolation, a comprehensive understanding of how human-machine collaboration specifically fosters digital creativity remains underdeveloped. This study deepens the exploration by revealing that digital creativity in AI-enabled workplaces emerges not merely from individual creative capabilities or technological access, but from the synergistic interaction between human cognitive-affective processes and AI collaborative partnership. Specifically, this study provides empirical support at the organizational behavior level for the “human-machine symbiosis” theory in AI ethics. This theory emphasizes that the value of AI lies not in replacing humans but in augmenting human capabilities through collaboration (Brynjolfsson and McAfee, 2014). By demonstrating that human-machine collaboration enhances digital creativity through both cognitive pathways (technology acceptance enabling resource utilization) and affective pathways (positive emotion generating psychological momentum), this study reveals how symbiosis operates at the individual psychological level. AI functions as a cooperative “partner” rather than a mere instrument (Zhou et al., 2021)—one that helps employees unlock their innovative potential not simply by providing computational resources, but by creating conditions where cognitive recognition and emotional empowerment converge to fuel creative performance. This discovery shifts the “human-machine symbiosis” concept from the macrolevel of technological philosophy to the microlevel of individual creative behavior, providing concrete psychological mechanisms that explain how collaborative partnership translates into creative outcomes. By specifying that digital creativity requires both cognitive acceptance of AI capabilities and positive affective experiences from collaboration, this study addresses a critical gap in creativity research: the lack of theoretically grounded explanations for human-AI co-creativity in organizational contexts. Furthermore, this study expands interdisciplinary theoretical dialogue by offering new empirical evidence for technology empowerment theory in the field of technopsychology. Technology empowerment theory posits that technology can promote individual behaviors by increasing feelings of control, competence, and independence (Zammuto et al., 2007). This study advances this theoretical conversation by demonstrating that technology empowerment operates through specific psychological sequences—cognitive empowerment (via technology acceptance) precedes and enables affective empowerment (via positive emotions), which subsequently drives creative performance. This represents a shift from general technology empowerment to collaborative empowerment—the specific mechanism wherein human-AI partnership generates creative benefits. This aligns with Chou et al. (2025), who demonstrated that AR technology empowers teachers through positive emotions, and extends their findings by establishing a generalizable framework for human-machine collaborative creativity across contexts.

### 4.3 Practical insights

The empirical results of this study offer critical practical insights for managers aiming to effectively deploy AI technologies and stimulate employees' digital creativity during digital transformation.

First, when promoting AI applications, enterprises should transcend a purely functional focus to prioritize employees' emotional and cognitive experiences, establishing a ”human-centered“ collaboration system. At the technical level, organizations should prioritize ”usability-oriented“ AI tools to lower technical barriers and enhance technology acceptance. Interface designs should remain intuitive, minimizing complex workflows while providing personalized guidance to help employees master new tools rapidly, thereby improving overall production efficiency (Zhai and Liu, 2023). Regarding task allocation, enterprises must adhere to the ”human–machine complementarity“ principle, clearly delineating responsibilities. By assigning repetitive, computational, and low-value tasks to AI, employees can liberate cognitive resources for high-value creative activities. This is essential for achieving the ”resource gain“ effect of collaboration while reducing career anxiety. This approach has proven successful in fields like smart healthcare, where AI handles record recognition while doctors focus on complex diagnosis and patient communication—tasks requiring deep emotional and creative intelligence (Gou et al., 2024). Furthermore, because the alignment between AI tools and job requirements significantly enhances perceived usefulness (Huu, 2023), companies should deploy personalized application scenarios tailored to specific roles and innovation needs.

Second, the enabling effect of human–machine collaboration relies on systematic organizational support and institutional safeguards. In terms of human resources, enterprises should establish specialized AI support teams, such as operations engineers and data analysts, to maintain systems and provide technical consultations. Dedicated funding must also be allocated for technology procurement, employee training, and innovation project support. From an institutional perspective, management systems—including performance evaluation, training, and communication protocols—should be tailored to integrate collaboration into daily workflows. Performance metrics should shift from traditional KPI-centric models to incorporate indicators such as “human–machine collaboration efficiency” and “digital innovation outcomes.” Furthermore, firms should establish robust feedback mechanisms, gathering employee suggestions through surveys and workshops to adjust technology deployment strategies. Such engagement significantly impacts a company's successful adoption of AI-driven innovative management models (Cimino et al., 2024).

Third, companies must build a positive emotional and psychological support system to fuel the emotional drive behind digital creativity. Since positive emotions serve as a crucial mediator, their maintenance requires an optimized work environment and emotional incentive mechanisms. Organizations should foster an innovative atmosphere that “encourages exploration and tolerates failure,” providing a safe psychological space where employees feel free to experiment without fear of negative repercussions. This is vital, as maintaining intrinsic motivation requires external autonomy and positive feedback (Lo, 2024). Beyond material rewards, enterprises should implement emotional incentives that recognize outstanding digital creativity, creating a virtuous cycle where positive emotions drive innovation. Finally, to prevent psychological depletion, companies should offer specialized support, such as Employee Assistance Programs (EAPs), offering professional counseling and stress management. By reasonably managing workloads and preventing over-reliance on AI, managers can ensure employees maintain a healthy work-rest balance, thereby safeguarding the psychological resources necessary for sustained digital innovation.

### 4.4 Research limitations and future prospects

Although this study has made some headway in examining how human–machine cooperation is associated with employee digital creativity, there are still certain issues that need to be addressed in other studies. First, this study utilized cross-sectional multi-source survey data for analysis. While this approach can verify correlations between variables, it fails to establish temporal precedence or definitive causal inferences. Observed relationships could be bidirectional or influenced by unmeasured third variables (e.g., individual dispositional optimism). The chain mediation pathway should be interpreted as consistent with rather than demonstrating a causal sequence. Future studies should adopt longitudinal designs or experimental manipulations. Second, regarding common method bias. Although we employed procedural remedies—including supervisor-rated measures for digital creativity (rather than self-reports), counterbalanced question ordering, and anonymous survey administration—common method variance may still influence the observed relationships. Because technology acceptance, positive emotions, and human-machine collaboration were all measured through employee self-reports in a single survey, shared perceptual biases (e.g., employees' general affectivity or impression management tendencies) could artificially inflate the observed associations. While Harman's single-factor test indicated that no single factor accounted for the majority of variance, this test has limited sensitivity. Consequently, the true strength of the relationships between variables may differ from those reported, and future studies should incorporate objective measures of creativity (e.g., actual creative output evaluations, innovation counts) and temporal separation of measurements to mitigate this concern. Third, regarding sampling constraints. This study utilized a purposive sampling strategy combining online maximum variation sampling and offline visits to AI-adopting enterprises in four Chinese regions (Shanghai, Zhejiang, Anhui, and Jiangsu). While this approach ensured diversity across industries (intelligent manufacturing, transportation, medicine, and new sales), the sample is not statistically representative of all digital-intelligent enterprises in China or globally. The concentration in four economically developed eastern Chinese provinces may introduce regional bias, as these areas typically have higher digital infrastructure maturity and AI adoption rates compared to western or rural regions. Additionally, the sampling focused on enterprises that had already adopted AI systems, potentially excluding organizations struggling with AI implementation or those in early adoption stages. These sampling characteristics limit the generalizability of findings to different economic contexts, cultural settings, or organizational maturity levels regarding AI adoption. Fourth, this study revealed that the path coefficient from technology acceptance to positive emotion (β = 0.12) and the chain mediation effect were relatively weak, suggesting that their relationship may be influenced by moderating variables. Future research could introduce such moderators to construct a moderated mediation model.

## Data Availability

The raw data supporting the conclusions of this article will be made available by the authors, without undue reservation.
